# Arm Swing and External Load Reorganize Force–Time Strategy in Collegiate Basketball Players: Associations with Force Production Capacity

**DOI:** 10.3390/sports14090377

**Published:** 2026-09-01

**Authors:** Tzu-Han Chan, Chieh-Ying Chiang, Yi-Cheng Wu, Sung-Kai Lin

**Affiliations:** 1 Department of Athletic Training and Health, National Taiwan Sport University, Taoyuan 33325, Taiwan; 1140601@ntsu.edu.tw; 2 Department of Sports Training Science-Combats, National Taiwan Sport University, Taoyuan 33325, Taiwan; 3 Department of Sports Science Research, Taiwan Institute of Sports Science, Kaohsiung 81343, Taiwan; 4 Department of Sport Medicine, Landseed International Hospital, Taoyuan 32449, Taiwan; keanu.firefox@gmail.com (Y.-C.W.); linedbeng@landseed.com.tw (S.-K.L.)

**Keywords:** countermovement jump, arm swing, loaded jump, force–time strategy, isometric mid-thigh pull, rate of force development, muscle architecture, basketball

## Abstract

Background: Vertical jump force–time strategy describes how force is organized in time. This strategy provides performance-relevant information that jump height alone does not capture in basketball players. Arm swing and external load are opposing conditions rarely imposed on the same athletes. However, little is known about how they reorganize jump strategy, or whether force production capacity is associated with the response. Methods: A within-participant, repeated-measures design was used. Seventeen collegiate male basketball players performed the countermovement jump (CMJ), the arm-swing countermovement jump (CMJA), and the 20 kg loaded countermovement jump (LCMJ) on force platforms in a fixed order within one session. The isometric mid-thigh pull provided relative peak force and the rate of force development (RFD_250_). Vastus lateralis and lateral gastrocnemius architecture were assessed by ultrasonography. Linear mixed-effects models examined condition effects and capacity associations, with a condition-mean sensitivity analysis. Results: Relative to CMJ, CMJA increased jump height and prolonged contact and concentric times. In contrast, LCMJ reduced jump height and increased mean concentric force. No significant overall condition effect was found for gross peak force, and equivalence was not established. Relative isometric peak force was not significantly associated with jump height. The trial-level CMJA-by-RFD_250_ interaction was significant, but the corresponding interaction was not significant in the condition-mean model. No architecture–RFD_250_ correlation remained significant after correction for multiple comparisons. Conclusions: In this exploratory fixed-order design, the arm-swing and loaded conditions were associated with divergent force–time responses. Evidence for an overall condition effect on gross peak force was inconclusive, and the capacity associations require confirmation in larger, counterbalanced samples.

## 1. Introduction

Vertical jumping is fundamental to competitive basketball. Jump performance is associated with playing time and on-court efficiency in professional players [1]. The countermovement jump (CMJ) is also widely used to monitor neuromuscular status in athletes [2]. Jump height is the outcome most commonly reported. However, this single value summarizes the movement rather than revealing the strategy that produces it. In applied monitoring, resolving this strategy could refine how jump tests inform training decisions.

The CMJ unfolds through distinct phases, from unweighting and eccentric braking to concentric propulsion. These phases are identified from the force–time and force–velocity records [3]. Within this framework, several variables quantify how force is organized across these phases, rather than the peak force produced [4]. These include contact time, concentric time, eccentric displacement, vertical stiffness, and mean concentric force. In professional basketball players, such force–time metrics differ by playing position and competitive role, even when jump height is comparable [5,6]. Strategy and maximal force describe separable dimensions of the same movement: the temporal organization of force can change while peak force remains stable. Consequently, a given athlete can reorganize this temporal strategy when the mechanical demand of the task is altered.

The mechanical demand of the CMJ can be altered in two opposing directions. At the unloading end, an arm swing augments jump height. This gain is accompanied by a longer acceleration phase and greater propulsive impulse, with no clear change in peak force [7]. At the loading end, an external load reduces jump height and alters the temporal expression of the stretch–shortening cycle [8,9]. This cycle depends on the coupling between eccentric and concentric phases. Each condition has been well characterized in isolation. However, the two have rarely been imposed on the same athletes and directly contrasted within a common force–time framework. This contrast is informative, because the conditions pull strategy in opposite directions and thereby isolate the temporal dimension from maximal force.

Existing work has emphasized the change in jump height produced by each condition, rather than the strategy through which that change arises. Furthermore, maximal isometric strength is associated with vertical jump performance in basketball players [10]. Isometric mid-thigh pull performance predicts jump and sprint outcomes in this population [11]. The rate-dependent expression of strength is governed by partly distinct mechanisms [12]. However, whether force production capacity is associated with the condition-specific response remains unknown.

This study aimed to (1) compare force–time strategy among the CMJ, the arm-swing condition (CMJA), and the loaded condition (LCMJ); (2) examine whether session-concurrent isometric force production capacity was associated with jump height and the condition-specific responses; and (3) examine whether muscle architecture was associated with rapid-force capacity in collegiate basketball players. It was hypothesized that (1) the arm-swing and loaded conditions would reorganize the force–time strategy in divergent ways, altering jump height and the distribution of force with little change in peak force, and (2) relative isometric peak force would be associated with jump height across conditions and rapid-force capacity would be associated with the arm-swing response.

## 2. Materials and Methods

### 2.1. Study Design

A within-participant, repeated-measures design was used. The conditions were performed in a fixed order. This kept any residual potentiation or fatigue consistent across participants, and recovery intervals between conditions reduced these effects. The order was: the countermovement jump with arm swing (CMJA), the standard countermovement jump (CMJ), the 20 kg loaded countermovement jump (LCMJ), and finally the isometric mid-thigh pull (IMTP). At least five minutes of recovery separated consecutive conditions. The 20 kg load was chosen to reproduce an established absolute-load protocol [8]. The 20 kg load corresponded to 24.4 ± 3.8% of body mass, with an individual range of 17.9% to 33.4%.

Testing took place between 08:00 and 12:00. This assessment formed part of the squad’s routine monitoring program. All participants were therefore familiar with every jump condition, including the loaded countermovement jump. Standardized warm-up and submaximal trials preceded testing, following established protocols [8,13].

### 2.2. Participants

Seventeen trained (Tier 3) [14] male basketball players competing at the collegiate level were recruited for this study (age: 20.4 ± 1.2 years; height: 184.6 ± 8.3 cm; body mass: 82.6 ± 12.0 kg; body mass index: 24.6 ± 1.8 kg·m^−2^). The Tier 3 (highly trained) classification was assigned on the basis of regular competition in the national collegiate league together with structured, sport-specific training [14]. Each player had accumulated at least six years of sport-specific training. Eligibility was restricted to athletes engaged in active collegiate competition. Players were excluded if they reported a current lower-body musculoskeletal injury or had undergone orthopedic surgery in the previous 12 months. Written informed consent was obtained from every participant before testing. Ethical approval was granted by the Institutional Review Board of Fu Jen Catholic University (C108178), and all procedures adhered to the Declaration of Helsinki.

### 2.3. Vertical Jump Testing

#### 2.3.1. Warm-Up and Familiarization

After the ultrasound assessment, a standardized warm-up was completed. Five minutes of low-intensity aerobic exercise were performed first, followed by dynamic mobility drills comprising bodyweight squats, walking lunges, and leg swings. Familiarization with each jump condition was provided immediately before that condition through two submaximal attempts performed at approximately 50% and 75% of perceived maximal effort.

#### 2.3.2. Jump Conditions

Three jump conditions were examined: CMJ, CMJA, and LCMJ. For the CMJ, the hands were kept on the hips throughout the movement, so that the arms made no contribution [7]. For the CMJA, the arms were free to swing; the action began with backward movement at the shoulder and continued into a forward swing, reproducing the athletes’ habitual jumping technique [7]. For the LCMJ, a 20 kg barbell was supported across the upper trapezius, between the seventh cervical and third thoracic vertebrae [8].

#### 2.3.3. Jump Execution

Every jump started from a still, upright stance, and the depth of the countermovement was self-selected. Athletes were asked to descend without pausing and to project themselves upward with maximal effort [7,8]. During the CMJA, the arm action accompanied the downward and upward phases and continued until the feet left the platform [7]. During the CMJ and LCMJ, the hand-on-hip posture and the barbell position, respectively, were maintained throughout the movement.

#### 2.3.4. Testing Protocol

One minute of recovery separated individual attempts. Two maximal trials were retained for each condition, and their mean represented that condition in the descriptive comparisons [8].

#### 2.3.5. Instrumentation and Data Processing

Ground reaction forces were sampled at 1000 Hz from a pair of force platforms (9260AA, Kistler, Winterthur, Switzerland). Signals were routed through an analog-to-digital converter (5695B, Kistler, Winterthur, Switzerland) and acquired with Bioware software (2812A, Kistler, Winterthur, Switzerland). The raw vertical force–time signal was stored and exported as text files for processing. Force–time variables were then computed for each jump condition within a purpose-built Microsoft Excel workbook (version 2016; Microsoft Inc., Redmond, WA, USA), following an established phase-analysis framework [3]. The same force platform and acquisition chain recorded the isometric mid-thigh pull, and the isometric force–time variables were likewise derived from the raw vertical signal. The definition and method of calculation for each variable are summarized in Table 1.

Within each jump, the movement between onset and take-off was partitioned to characterize the force–time strategy [3]. The net impulse was the vertical impulse over this whole interval. The eccentric impulse was the net impulse of the braking phase, from the instant force returned to bodyweight until velocity reached zero. The concentric impulse was the net impulse of the propulsion phase, from zero velocity to take-off. No filtering or smoothing was applied, because unfiltered data are recommended for onset-dependent variables [3,13]. Bodyweight was the mean vertical force over a quiet-standing weighing period of at least 1 s [3,4]. For the loaded condition, this quantity was the system weight of the participant and the 20 kg bar, and system mass was used throughout [4]. Net force was obtained by subtracting this weight. Net force was then integrated by the trapezoidal rule to yield velocity and by velocity to yield displacement [3,4]. Power was the product of force and velocity at each sample [4]. No post hoc correction for integration drift was applied. Integration commenced during the quiet-standing weighing period, with initial velocity and displacement set to zero [3].

Take-off was defined as the first sample at which vertical force fell below five standard deviations of the flight-phase force, and touchdown as the first subsequent sample at which vertical force exceeded the same threshold [3].

The outcome variables were jump height and the RSI-mod. Jump height was calculated from take-off velocity as v-squared divided by twice the gravitational acceleration [3]. The force–time strategy was characterized by the net, eccentric, and concentric impulses defined above, together with additional strategy variables [3,4]. These further comprised the phase durations, peak and mean concentric forces, and vertical stiffness. They also comprised peak and take-off velocity, eccentric displacement, and mean concentric and peak power. All reported impulses are net values. Peak force values are gross and include bodyweight.

### 2.4. Skeletal Muscle Architecture Assessment

#### 2.4.1. Ultrasonographic Imaging

The vastus lateralis and lateral gastrocnemius were selected because their architectural characteristics have previously been associated with lower-body strength, power, and muscle–tendon stiffness [16,17]. Resting architecture of the lower-limb muscles was imaged at the start of the session, before the warm-up and any jumping, using a B-mode ultrasound device (E-CUBE 8 DIAMOND, Alpinion Medical Systems, Seoul, Republic of Korea) fitted with a 3–12 MHz linear transducer. All scans were acquired by a single physician who was blinded to the jump and isometric performance data. Two images were obtained for each muscle on each limb and averaged within limb. The left- and right-limb values were then averaged to obtain a bilateral mean. Fascicle length was estimated separately for each limb before bilateral averaging [16,17].

#### 2.4.2. Vastus Lateralis (VL)

For the vastus lateralis, participants lay supine with their legs fully extended and relaxed. The transducer was aligned along the muscle at the midpoint between the greater trochanter and the lateral epicondyle of the femur [16,17]. Contact pressure was kept to a minimum to avoid compression of the underlying tissue.

#### 2.4.3. Lateral Gastrocnemius (LG)

For the lateral gastrocnemius, participants lay prone with the ankle relaxed in a neutral position. The transducer was aligned along the muscle at two-thirds of the distance from the lateral epicondyle of the femur to the lateral malleolus [16,17].

#### 2.4.4. Image Analysis

Image measurements were performed in ImageJ (version 1.53v, National Institutes of Health, Bethesda, MD, USA) by the physician who had collected the scans. Muscle thickness was taken as the perpendicular separation between the superficial and deep aponeuroses. Pennation angle was taken as the angle formed between a fascicle and the deep aponeurosis. Fascicle length was estimated as muscle thickness divided by the sine of the pennation angle [17].

### 2.5. Isometric Mid-Thigh Pull

After the jump conditions, isometric mid-thigh pull (IMTP) testing was performed on the same force platform [15]. An immovable bar was set at the mid-thigh, just below the crease of the hip, and its height was adjusted for each participant. The isometric mid-thigh pull was selected because it provides peak force and epoch-specific rate of force development from a single maximal effort. Compared with repetition-maximum testing, it is less fatiguing and carries a lower injury risk, which suits repeated in-season monitoring [13]. Bar height was set so each participant adopted an upright trunk, with a knee angle of 125 to 145 degrees and a hip angle of 140 to 150 degrees, as recommended previously [13]. This replicates the start of the second pull of the clean [13,15]. The hands were strapped to the bar [15]. After the stance had stabilized, the cue “3, 2, 1, Pull” was delivered, with only minimal pretension permitted to remove any slack before the effort began [15]. Trials showing a countermovement, excessive pretension, or leaning on the bar were rejected and repeated [13]. Each trial lasted five seconds and required the participant to pull against the bar as forcefully and as rapidly as possible.

IMTP force onset was defined as the first sample at which vertical force exceeded the mean bodyweight baseline, determined during the weighing period, by five standard deviations [13]. The highest force recorded during the trial defined peak force, and dividing this value by body mass yielded relative peak force [15]. The rate of force development was quantified across the first 250 ms after force onset (RFD_250_) from the force–time record [15]. Two maximal trials were performed. Trials were separated by at least three minutes to avoid fatigue [15]. A further trial was collected if the two peak forces differed by more than 250 N [13]. When a third trial was required, the two trials with the closest peak forces were retained. Relative peak force and RFD_250_ were calculated for each retained trial, and the mean of the two retained trials was used in the condition-mean analyses and correlations. Both retained trials were entered separately in the trial-level models. The 0–250 ms epoch was selected before inferential modeling. The 250 ms window most closely approximated the propulsion-phase duration observed in this cohort, and met the pre-specified reliability criteria.

### 2.6. Statistical Analysis

Statistical analyses were conducted in R (version 4.3.3; R Foundation for Statistical Computing, Vienna, Austria). Reliability was assessed between the two retained trials of each jump and isometric variable. For architecture variables, it was assessed between the two images acquired per muscle. Three indices were computed. The intraclass correlation coefficient (ICC) used here was ICC(2, k), a two-way random-effects, absolute-agreement, average-measures coefficient. This form was specified because the analysis units were two-trial condition means rather than single trials. The standard error of measurement (SEM) was the typical error. This was the standard deviation of the trial-to-trial difference divided by the square root of two. The coefficient of variation (CV) expressed that typical error relative to the grand mean. The SEM and CV therefore represent the typical error of a single trial, whereas ICC(2, k) represents the reliability of the mean of the two trials. Variables with an ICC below 0.70 or a CV above 10% were excluded [18,19]. Every variable assessed is reported in Table 2. No a priori sample-size calculation was performed. The sample comprised the full available roster of a single collegiate squad assessed within one monitoring session. The association and moderation analyses are therefore exploratory, and were not preregistered.

Degrees of freedom were obtained by Satterthwaite approximation as implemented in lmerTest (version 3.1-3). For the balanced design used here, these correspond to 67 for within-participant terms in the trial-level models. They correspond to 32 for within-participant terms and 15 for between-participant terms in the condition-mean models. Model diagnostics extended beyond the Shapiro–Wilk test of residuals. Residuals were plotted against fitted values, and homogeneity of residual variance across conditions was examined. Influence was examined by refitting each model with each participant omitted in turn.

Paired effect sizes were expressed as Hedges’ *g*, with 95% confidence intervals estimated using 5000 bootstrap resamples of within-subject differences. Linear mixed-effects models were fitted to trial-level data using the lme4 (version 1.1-35.1) and lmerTest (version 3.1-3) packages to examine condition effects and the moderating role of neuromuscular capacity. Each jump trial provided one observation, giving 102 trial-level observations (17 participants × 3 conditions × 2 trials). The paired predictor was the participant’s mean IMTP RFD_250_, calculated from their two retained IMTP trials. Condition was specified as a fixed effect and Subject_ID as a random intercept, with CMJ set as the reference condition. Continuous IMTP predictors were standardized as z-scores. The first model examined the association between relative IMTP peak force and jump height across conditions. The second model examined whether IMTP RFD_250_ moderated condition-specific jump-height responses through condition-by-RFD_250_ interaction terms; this model was specified as jump height ~ condition × RFD_250_ + (1 | participant), with RFD_250_ standardized. A by-participant random slope for condition was examined as a robustness check. As a sensitivity analysis, the models were refitted on each participant’s two-trial condition means to address the non-independence of trial-level observations. Residual normality was confirmed (Shapiro–Wilk *p* > 0.05). Pairwise condition comparisons were Holm-adjusted.

Pearson correlations were used to examine subject-level associations between muscle architecture and IMTP-derived capacity, with confidence limits derived using Fisher’s z transformation. Six architecture–RFD_250_ correlations were analyzed as a single pre-specified family with false-discovery-rate control under the architectural gearing framework. The Benjamini–Hochberg false discovery rate was then applied across this family. Correlation magnitudes were interpreted as negligible (|*r*| < 0.10), weak (0.10–0.39), moderate (0.40–0.69), strong (0.70–0.89), and very strong (≥ 0.90) [20]. Statistical significance was set at *p* < 0.05.

## 3. Results

### 3.1. Measurement Reliability and Within-Condition Trial Comparisons

All retained force–time variables and IMTP-derived measures showed acceptable reliability, with average-measures ICC values of 0.81 or greater and CV at or below 6.0% (Table 2). Within-jump rate of force development and time to peak force were excluded from analysis because their CV exceeded 10% in at least one unloaded condition.

Phase-specific impulse and power variables were reliable in all three conditions. Average-measures ICC values were 0.939 or greater, and coefficients of variation were 5.5% or lower (Table 2). All ultrasound-derived architecture variables met the pre-specified thresholds (ICC ≥ 0.70 and CV ≤ 10%). Among the isometric epochs, the 0–200 ms and 0–250 ms windows met the thresholds, whereas the 0–50 ms and 0–100 ms windows did not.

### 3.2. Condition Effects on Jump Strategy

Jump strategy differed across conditions (Table 3). Relative to CMJ, CMJA increased jump height (*g* = 0.86) and prolonged contact and concentric times (*g* = 2.09 and 1.27, respectively). During CMJA, vertical stiffness was reduced (*g* = −0.60). Relative to CMJ, LCMJ reduced jump height (*g* = −1.77) and the RSI-mod (*g* = −2.04). Mean concentric force was increased under load (*g* = 0.46). Vertical stiffness during LCMJ did not differ from CMJ (*g* = 0.07). No significant overall condition effect was found for gross peak force (*p* = 0.076). However, the estimates did not establish equivalence, and the loaded condition showed a small effect relative to CMJ (*g* = 0.24, 95% CI: 0.09 to 0.50).

**Figure 1:**
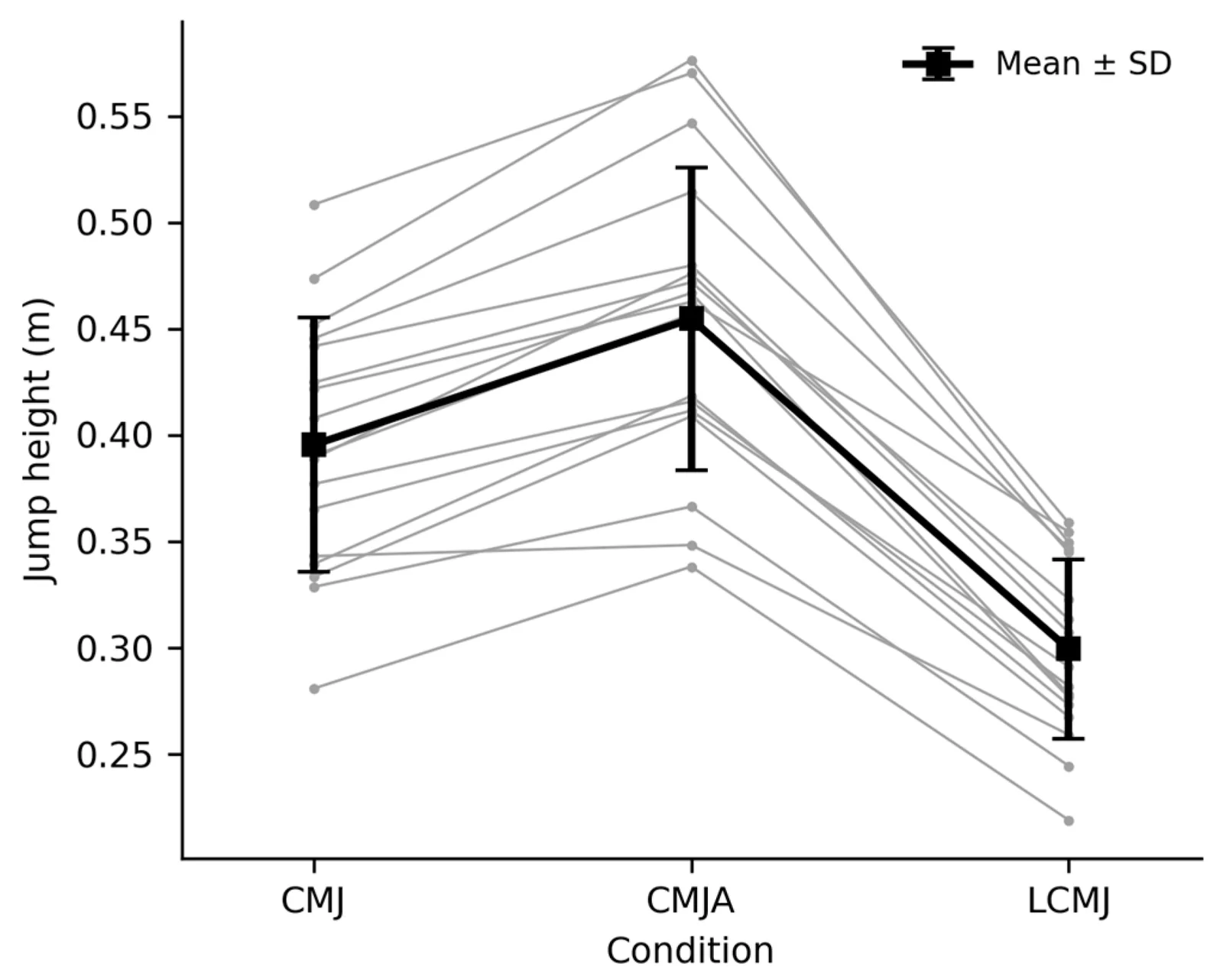
Individual jump-height responses across the three conditions (*n* = 17). Gray lines represent individual participants. The black line represents the group mean ± SD.

Phase-specific impulse and power also differed across conditions (Table 3). The net impulse was greater during both CMJA and LCMJ than during CMJ. The concentric impulse showed the same pattern and did not differ between the two manipulated conditions. Under load, the eccentric impulse of the braking phase was increased. Mean concentric power was reduced under load. During CMJA, peak power was increased relative to CMJ, whereas mean concentric power was unchanged. Individual responses across the three conditions are presented in Figure 1.

The full coefficients for the trial-level and condition-mean specifications of both models are reported in Table 4. Residual variance differed across conditions in the trial-level model, but not in the condition-mean model. Omitting any single participant changed the CMJA-by-RFD_250_ interaction coefficient by no more than 0.0022 m per standard deviation.

### 3.3. Neuromuscular Capacity and Condition-Specific Responses

**Figure 2:**
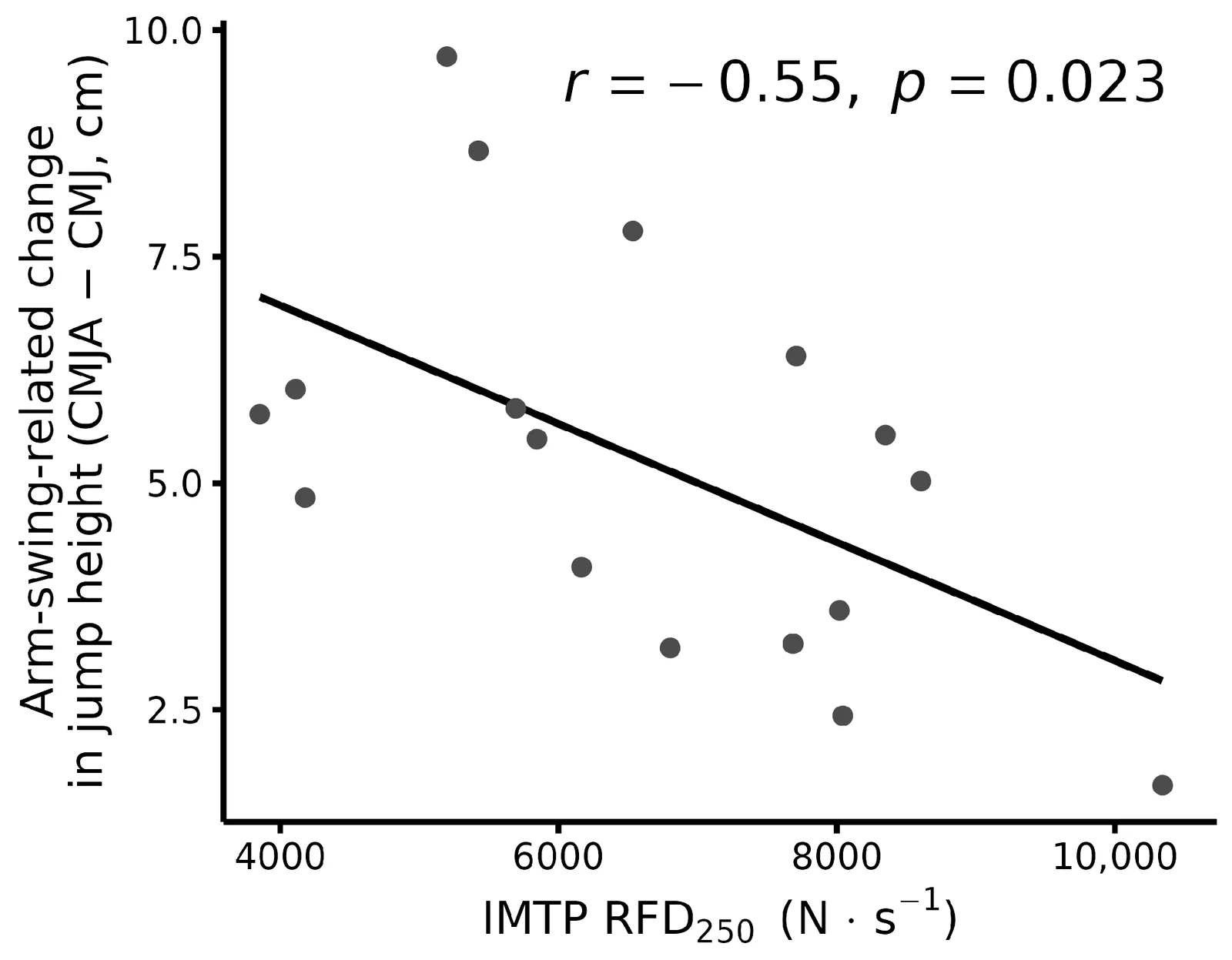
The fitted line and Pearson correlation are presented descriptively. Inferential interpretation is based on the trial-level and condition-mean mixed-effects models reported in Table 4.

Relative IMTP peak force was not significantly associated with jump height in either the trial-level or condition-mean model (*β* = 1.38 cm per SD, 95% CI [−1.62, 4.38], *p* = 0.342; Table 4). IMTP RFD_250_ was not associated with CMJ height (*p* = 0.965). The CMJA-by-RFD_250_ interaction was significant in the trial-level model (*β* = −1.24 cm per SD, *p* = 0.012), but did not reach statistical significance in the condition-mean model (*p* = 0.099; Figure 2). No LCMJ-by-RFD_250_ interaction was found in either specification (*p* = 0.682 and 0.788; Table 4). The random-slope model was singular and did not converge, so the random-intercept specification was retained.

### 3.4. Muscle Architecture and Neuromuscular Capacity

No architecture–RFD_250_ correlation remained significant after Benjamini–Hochberg correction. Before correction, vastus lateralis muscle thickness and fascicle length showed moderate positive correlations with IMTP RFD_250_ (*r* = 0.57 and 0.48, respectively; Table 5). The corresponding correlations with relative IMTP peak force were smaller (*r* = 0.20 and 0.41). Lateral gastrocnemius pennation angle was not significantly associated with either capacity measure.

## 4. Discussion

The present study aimed to compare force–time strategy across CMJ, CMJA, and LCMJ and to examine associations with session-concurrent IMTP-derived force capacity. The arm-swing and loaded conditions were accompanied by divergent changes in jump height, phase duration, impulse, and power. Relative IMTP peak force was not associated with jump height, and the CMJA-by-RFD_250_ interaction was not confirmed in the condition-mean analysis. Overall, the findings suggest that multi-condition testing captures distinct jump responses, although the capacity associations remain exploratory.

The arm-swing condition increased jump height. Contact and concentric times lengthened, and vertical stiffness decreased. These changes are consistent with previous reports that arm swing prolongs the acceleration phase and increases propulsive impulse [7]. However, the present data do not determine whether these temporal changes caused the increase in jump height. The arm-swing condition was accompanied by changes in temporal structure, whereas evidence for a change in gross peak force remained inconclusive.

The loaded condition reduced jump height and the RSI-mod. Mean concentric force increased. These findings are consistent with the possibility that an external load constrains the rapid expression of the stretch–shortening cycle and shifts the movement toward a slower, concentrically dominant strategy [8,9]. The eccentric phase may contribute to the subsequent concentric output [21]. The reduced reactive performance under load is consistent with a constrained stretch–shortening cycle [22]. Both conditions therefore prolonged the movement and lengthened contact and concentric times. The opposition lay in how force was distributed within that lengthened time. The arm-swing condition was accompanied by lower stiffness and concentric force and by greater jump height. In contrast, the load raised concentric force and reduced reactivity as jump height fell.

Equivalence was not established, and evidence for an overall condition effect on gross peak force was inconclusive. Condition differences were more consistently observed in phase duration, impulse, and power than in gross peak force. A focus on jump height or peak force alone would therefore have obscured the force–time differences between conditions. This interpretation is consistent with previously reported position- and role-related differences in basketball force–time metrics [6]. Relative isometric peak force was not significantly associated with jump height in either model. The present findings therefore did not support an association between session-concurrent relative isometric peak force and jump height across conditions [10].

The rate-dependent component behaved differently. Rapid-force expression is governed by mechanisms that are partly distinct from those governing maximal force [12]. Early-phase rate of force development is comparable between isometric and dynamic muscle actions [23]. A static isometric pull therefore provides a complementary, session-concurrent index of rapid-force capacity. The trial-level model showed a negative CMJA-by-RFD_250_ interaction. However, this interaction was not significant in the condition-mean model and is therefore interpreted as exploratory. No corresponding interaction was found for LCMJ. Because the IMTP followed all jump conditions, the preceding activity may also have influenced RFD_250_.

No architecture–RFD_250_ correlation remained significant after false-discovery-rate correction. The unadjusted associations involving vastus lateralis muscle thickness and fascicle length are therefore considered hypothesis-generating [24,25,26,27,28,29].

This study is not without limitations. First, the sample comprised 17 players from one collegiate squad, which limited statistical power for the association and interaction analyses and restricts generalization to other populations. Second, the fixed order of conditions is a design limitation. Because every participant completed the conditions in the same sequence, condition effects are confounded with order, and the within-condition trial comparison could not be resolved. Third, the IMTP followed all jump conditions, and the preceding activity may have influenced RFD_250_. Fourth, the fixed 20 kg load produced different relative stimuli among athletes and limits generalization to other relative loads. Fifth, no significant overall condition effect was found for gross peak force, but equivalence was not established. Sixth, individual knee and hip angles within the specified IMTP ranges were not recorded, limiting the assessment of between-participant variation in position. Finally, fascicle length was estimated rather than measured directly, and architecture was assessed only in the vastus lateralis and lateral gastrocnemius.

## 5. Practical Applications

The arm-swing and loaded jumps may provide complementary information about competition-specific coordination and responses to added mechanical demand. Whether both conditions are included depends on the monitoring aim. Individual responses, including the exploratory RFD_250_ pattern, require cautious interpretation. Future studies could incorporate continuous force–time analyses, together with architectural measures spanning additional muscle groups, across larger and more diverse samples [30,31,32].

## 6. Conclusions

This study compared arm-swing and loaded countermovement jumps within a single cohort of collegiate male basketball players. In this fixed-order design, the conditions were accompanied by divergent changes in jump height, phase duration, impulse, and power. Evidence for an overall condition effect on gross peak force was inconclusive, and relative IMTP peak force was not significantly associated with jump height. The CMJA-by-RFD_250_ interaction was significant only in the trial-level model and was not confirmed in the condition-mean analysis. Multi-condition jump testing may provide complementary information beyond jump height alone. These findings may warrant confirmation in larger, counterbalanced, and prospectively powered samples.

## Figures and Tables

**Table 1:** Definitions and methods of calculation for the force–time and isometric variables.

Variable	Definition and Method of Calculation	Source
Jump height (JH)	Derived from the vertical take-off velocity using the impulse–momentum method, as JH = v^2^/(2g), where v is the vertical velocity of the center of mass at take-off, obtained by integrating the net vertical force divided by body mass, and g is gravitational acceleration (m).	[3,4]
Reactive strength index-modified (RSI-mod)	Jump height divided by the time to take-off (RSI-mod = JH/time to take-off).	[3]
Contact time (ContactT)	Time from the onset of the countermovement to take-off; onset defined as the instant the vertical force departed from the quiet-standing value beyond a fixed threshold (s).	[3]
Concentric time (ConcT)	Duration of the propulsion (concentric) phase, from the lowest point of the center of mass to take-off (s).	[3]
Eccentric displacement (EccDisp)	Downward displacement of the center of mass during the countermovement, from quiet-standing to its lowest point, obtained by double integration of the vertical acceleration (cm).	[3,4]
Peak force (PF)	Maximum vertical ground reaction force recorded during the movement (N).	[4]
Mean concentric force (MCF)	Mean vertical ground reaction force during the propulsion (concentric) phase (N).	[3]
Net impulse (NetImp)	Net vertical impulse over the entire movement, from the onset of the countermovement to take-off, obtained as the time integral of the force minus bodyweight. It determines take-off velocity (N·s).	[3,4]
Eccentric impulse (EccImp)	Net vertical impulse during the braking phase, from the instant force returns to bodyweight until the center-of-mass velocity reaches zero (N·s).	[3,4]
Concentric impulse (ConImp)	Net vertical impulse during the propulsion phase, from zero velocity to take-off (N·s).	[3,4]
Peak velocity	Peak instantaneous vertical velocity of the center of mass (m·s^−1^).	[4]
Take-off velocity	Vertical velocity of the center of mass at the instant of take-off (m·s^−1^).	[3,4]
Vertical stiffness (Kvert)	Ratio of the peak vertical force to the corresponding vertical displacement of the center of mass during the countermovement (kN·m^−1^).	[3]
Mean concentric power (MCP)	Mean mechanical power during the propulsion phase, obtained as the product of vertical force and center-of-mass velocity (W).	[4]
Peak power (PP)	Peak instantaneous mechanical power applied to the center of mass (W).	[4]
Isometric peak force (PF)	Highest vertical force recorded during the five-second maximal isometric mid-thigh pull; relative peak force expressed per kilogram of body mass (N or N·kg^−1^).	[4,15]
Rate of force development (RFD_250_)	Rate of rise in vertical force over the first 250 ms from force onset (Δforce/Δtime; N·s^−1^).	[15]

Variables were derived from the raw vertical ground reaction force sampled at 1000 Hz. Center-of-mass velocity and displacement were obtained by forward-dynamics (impulse–momentum) integration of the net vertical force, and movement phases (unweighting, braking, and propulsion) were defined according to the analytical framework of McMahon et al. [3]. Movement onset was identified when the vertical force deviated from the quiet-standing bodyweight by more than five standard deviations; take-off was identified when the vertical force fell below five standard deviations of the flight-phase force [3]. g denotes gravitational acceleration. The upper rows describe the vertical jump conditions; the final two rows describe the isometric mid-thigh pull.

**Table 2:** Within-session reliability of the force–time, isometric, and muscle architecture variables.

Variable	Cond.	Mean ± SD	ICC(2, k) [95% CI]	SEM	CV (%)
Jump variables					
Jump height (m)	CMJ	0.40 ± 0.06	0.985 [0.92, 0.99]	0.011	2.7
	CMJA	0.46 ± 0.07	0.990 [0.95, 0.99]	0.010	2.2
	LCMJ	0.30 ± 0.04	0.985 [0.91, 0.99]	0.007	2.2
RSI-mod	CMJ	0.52 ± 0.09	0.968 [0.84, 0.98]	0.023	4.3
	CMJA	0.52 ± 0.09	0.983 [0.90, 0.99]	0.015	3.0
	LCMJ	0.36 ± 0.06	0.944 [0.74, 0.96]	0.019	5.3
Contact time (s)	CMJ	0.76 ± 0.06	0.813 [0.49, 0.93]	0.034	4.5
	CMJA	0.89 ± 0.06	0.927 [0.78, 0.97]	0.021	2.4
	LCMJ	0.83 ± 0.05	0.829 [0.52, 0.94]	0.028	3.4
Concentric time (s)	CMJ	0.26 ± 0.03	0.896 [0.72, 0.96]	0.012	4.5
	CMJA	0.29 ± 0.02	0.954 [0.88, 0.98]	0.007	2.5
	LCMJ	0.30 ± 0.02	0.842 [0.56, 0.94]	0.013	4.3
Eccentric displacement (cm)	CMJ	39.3 ± 4.1	0.878 [0.66, 0.96]	2.09	5.3
	CMJA	44.1 ± 6.3	0.970 [0.91, 0.99]	1.40	3.2
	LCMJ	39.9 ± 3.6	0.853 [0.59, 0.95]	1.97	4.9
Peak force (N)	CMJ	2063 ± 216	0.979 [0.94, 0.99]	45.2	2.2
	CMJA	2085 ± 286	0.980 [0.95, 0.99]	58.2	2.8
	LCMJ	2120 ± 228	0.974 [0.93, 0.99]	51.9	2.4
Mean concentric force (N)	CMJ	1751 ± 187	0.982 [0.95, 0.99]	36.5	2.1
	CMJA	1695 ± 204	0.995 [0.99, 1.00]	20.5	1.2
	LCMJ	1844 ± 196	0.980 [0.94, 0.99]	40.8	2.2
Net impulse (N·s)	CMJ	242.1 ± 30.4	0.997 [0.99, 1.00]	2.49	1.0
	CMJA	257.6 ± 32.9	0.998 [0.99, 1.00]	2.33	0.9
	LCMJ	258.8 ± 30.8	0.996 [0.98, 1.00]	2.63	1.0
Eccentric impulse (N·s)	CMJ	136.2 ± 21.5	0.977 [0.86, 0.98]	4.25	3.1
	CMJA	129.6 ± 26.0	0.978 [0.88, 0.98]	5.32	4.1
	LCMJ	157.6 ± 25.1	0.974 [0.87, 0.98]	5.79	3.7
Concentric impulse (N·s)	CMJ	239.7 ± 29.9	0.996 [0.98, 1.00]	2.71	1.1
	CMJA	254.9 ± 32.6	0.998 [0.99, 1.00]	2.20	0.9
	LCMJ	255.0 ± 30.2	0.995 [0.97, 1.00]	2.75	1.1
Peak velocity (m·s^−1^)	CMJ	2.90 ± 0.20	0.988 [0.94, 0.99]	0.031	1.1
	CMJA	3.10 ± 0.23	0.993 [0.96, 0.99]	0.028	0.9
	LCMJ	2.56 ± 0.16	0.983 [0.90, 0.99]	0.026	1.0
Take-off velocity (m·s^−1^)	CMJ	2.78 ± 0.21	0.986 [0.93, 0.99]	0.036	1.3
	CMJA	2.98 ± 0.24	0.990 [0.95, 0.99]	0.034	1.1
	LCMJ	2.42 ± 0.17	0.985 [0.91, 0.99]	0.027	1.1
Vertical stiffness (kN·m^−1^)	CMJ	53.0 ± 6.7	0.901 [0.72, 0.96]	3.07	5.8
	CMJA	48.0 ± 8.8	0.945 [0.85, 0.98]	2.83	5.9
	LCMJ	53.4 ± 6.5	0.905 [0.74, 0.97]	2.87	5.4
Mean concentric power (W)	CMJ	1364 ± 171	0.949 [0.76, 0.96]	55.8	4.1
	CMJA	1379 ± 222	0.987 [0.93, 0.99]	36.1	2.6
	LCMJ	1109 ± 171	0.939 [0.71, 0.96]	60.8	5.5
Peak power (W)	CMJ	4537 ± 630	0.990 [0.95, 0.99]	90.7	2.0
	CMJA	5254 ± 777	0.992 [0.95, 0.99]	92.5	1.8
	LCMJ	4377 ± 614	0.991 [0.95, 0.99]	84.2	1.9
Jump rate of force development (N·s^−1^)	CMJ	7406 ± 2258	0.930 [0.68, 0.95]	837.6	11.3
	CMJA	3742 ± 2327	0.966 [0.83, 0.98]	596.3	15.9
	LCMJ	5538 ± 1478	0.939 [0.71, 0.96]	498.3	9.0
Jump time to peak force (s)	CMJ	0.19 ± 0.09	0.719 [0.14, 0.81]	0.065	33.8
	CMJA	0.40 ± 0.10	0.981 [0.90, 0.99]	0.020	5.2
	LCMJ	0.22 ± 0.08	0.992 [0.95, 0.99]	0.009	4.2
Isometric variables					
Isometric peak force (N)		3184 ± 631	0.992 [0.98, 1.00]	77.6	2.4
Relative isometric peak force (N·kg^−1^)		38.12 ± 5.41	0.986 [0.96, 0.99]	0.90	2.4
RFD (0–250 ms) (N·s^−1^)		6621 ± 1806	0.977 [0.94, 0.99]	398.5	6.0
RFD (0–100 ms) (N·s^−1^)		7089 ± 2124	0.690 [0.07, 0.80]	1694.8	23.9
RFD (0–50 ms) (N·s^−1^)		4746 ± 1718	0.583 [−0.09, 0.74]	1596.9	33.7
Muscle architecture					
VL muscle thickness (cm)		2.72 ± 0.27	0.997 [0.99, 1.00]	0.019	0.7
VL pennation angle (°)		22.51 ± 2.60	0.956 [0.79, 0.97]	0.752	3.3
VL fascicle length (cm)		7.19 ± 0.56	0.916 [0.63, 0.94]	0.229	3.2
LG muscle thickness (cm)		1.32 ± 0.26	0.999 [0.99, 1.00]	0.012	0.9
LG pennation angle (°)		13.77 ± 1.96	0.964 [0.82, 0.97]	0.538	3.9
LG fascicle length (cm)		5.56 ± 0.81	0.967 [0.83, 0.98]	0.213	3.8

CMJ, countermovement jump; CMJA, countermovement jump with arm swing; LCMJ, loaded countermovement jump; IMTP, isometric mid-thigh pull; RSI-mod, reactive strength index-modified; RFD, rate of force development; SEM, standard error of measurement; CV, coefficient of variation. ICC values are average-measures coefficients (two-way random-effects, absolute-agreement), consistent with the two-trial condition means used in the analysis. Within-condition trial comparisons were examined by comparing the first and second retained trials within each condition (Table S1). Of the 53 comparisons, six reached statistical significance, all with small changes (|Δ| ≤ 3.3%) and inconsistent in direction.

**Table 3:** Means ± SD, overall linear mixed-model effects, and paired Hedges’ *g* effect sizes for jump performance and force–time variables across CMJ, CMJA, and LCMJ conditions.

Variable	CMJ Mean ± SD	CMJA Mean ± SD	LCMJ Mean ± SD	*p*-Value (LMM)	CMJA vs. CMJ Hedges’ *g* [95% CI]	LCMJ vs. CMJ Hedges’ *g* [95% CI]	LCMJ vs. CMJA Hedges’ *g* [95% CI]
JH (m)	0.40 ± 0.06	0.46 ± 0.07 *^^^	0.30 ± 0.04 ^#^^	<0.001	0.86 [0.66, 1.28]	−1.77 [−2.55, −1.42]	−2.53 [−3.65, −2.09]
RSI-mod	0.52 ± 0.09	0.52 ± 0.09 ^^^	0.36 ± 0.06 ^#^^	<0.001	−0.08 [−0.32, 0.14]	−2.04 [−2.97, −1.65]	−1.89 [−2.77, −1.53]
ContactT (s)	0.76 ± 0.06	0.89 ± 0.06 *^^^	0.83 ± 0.05 ^#^^	<0.001	2.09 [1.49, 3.28]	1.27 [0.83, 2.71]	−1.04 [−1.64, −0.67]
ConcT (s)	0.26 ± 0.03	0.29 ± 0.02 *	0.30 ± 0.02 ^#^	<0.001	1.27 [0.80, 2.34]	1.68 [1.07, 3.40]	0.36 [0.06, 0.87]
EccDisp (cm)	39.30 ± 4.14	44.13 ± 6.30 *^^^	39.94 ± 3.56 ^^^	<0.001	0.88 [0.57, 1.32]	0.16 [−0.07, 0.46]	−0.81 [−1.24, −0.46]
PF (N)	2063.16 ± 215.75	2084.83 ± 285.55	2119.57 ± 228.26	0.076	0.08 [−0.26, 0.45]	0.24 [0.09, 0.50]	0.13 [−0.21, 0.52]
MCF (N)	1751.11 ± 186.60	1694.77 ± 204.35 *^^^	1843.85 ± 196.35 ^#^^	<0.001	−0.27 [−0.60, −0.13]	0.46 [0.33, 0.82]	0.71 [0.49, 1.25]
NetImp (N·s)	242.11 ± 30.77	257.64 ± 33.41 *	258.79 ± 31.18 ^#^	<0.001	0.46 [0.35, 0.73]	0.51 [0.41, 0.79]	0.03 [−0.05, 0.12]
EccImp (N·s)	136.15 ± 21.51	129.64 ± 26.09 *^^^	157.57 ± 25.18 ^#^^	<0.001	−0.26 [−0.53, −0.04]	0.87 [0.65, 1.40]	1.04 [0.78, 1.67]
ConImp (N·s)	239.72 ± 30.29	254.91 ± 33.02 *	255.03 ± 30.63 ^#^	<0.001	0.46 [0.35, 0.74]	0.48 [0.37, 0.73]	0.00 [−0.09, 0.09]
MCP (W)	1363.71 ± 171.31	1378.95 ± 222.24 ^^^	1109.39 ± 170.81 ^#^^	<0.001	0.07 [−0.21, 0.28]	−1.42 [−2.26, −1.05]	−1.30 [−2.07, −1.05]
PP (W)	4537.33 ± 629.63	5254.21 ± 777.37 *^^^	4376.83 ± 614.31 ^#^^	<0.001	0.97 [0.78, 1.44]	−0.25 [−0.46, −0.13]	−1.19 [−1.95, −0.93]
Kvert (kN·m^−1^)	52.96 ± 6.72	48.04 ± 8.85 *^^^	53.42 ± 6.50 ^^^	<0.001	−0.60 [−1.10, −0.28]	0.07 [−0.20, 0.40]	0.67 [0.35, 1.25]

Values are mean ± SD from each participant’s two-trial condition mean (*n* = 17 per condition). Overall *p*-values are based on trial-level linear mixed-effects models (LMM) with condition as a fixed effect and Subject_ID as a random intercept. * CMJA differs from CMJ (*p* < 0.05); ^#^ LCMJ differs from CMJ (*p* < 0.05); ^^^ CMJA differs from LCMJ (*p* < 0.05); pairwise comparisons were Holm-adjusted. Hedges’ *g* is a bias-corrected paired effect size with 95% confidence intervals (CIs) from 5000 bootstrap resamples. CMJ, countermovement jump; CMJA, countermovement jump with arm swing; LCMJ, 20 kg loaded countermovement jump; JH, jump height; RSI-mod, reactive strength index-modified; ContactT, contact time; ConcT, concentric time; EccDisp, eccentric displacement; PF, peak force; MCF, mean concentric force; Kvert, vertical stiffness; NetImp, net impulse; EccImp, eccentric impulse; ConImp, concentric impulse; MCP, mean concentric power; PP, peak power. Impulses are net values over their respective phases.

**Table 4:** Trial-level and condition-mean mixed-effects model coefficients for the strength–capacity association (Model 1) and RFD_250_ moderation (Model 2).

Term	Specification	Estimate (m)	95% CI	t	*p*
Model 1: relative IMTP peak force					
Intercept (CMJ)	Trial-level	0.3954	[0.3676, 0.4232]	28.36	<0.001
	Condition-mean	0.3954	[0.3663, 0.4245]	27.65	<0.001
CMJA vs. CMJ	Trial-level	0.0592	[0.0494, 0.0690]	12.06	<0.001
	Condition-mean	0.0592	[0.0442, 0.0742]	8.02	<0.001
LCMJ vs. CMJ	Trial-level	−0.0960	[−0.1058, −0.0862]	−19.57	<0.001
	Condition-mean	−0.0960	[−0.1111, −0.0810]	−13.02	<0.001
Relative IMTP peak force (z)	Trial-level	0.0138	[−0.0162, 0.0438]	0.98	0.342
	Condition-mean	0.0138	[−0.0162, 0.0438]	0.98	0.342
Model 2: condition × RFD_250_					
Intercept (CMJ)	Trial-level	0.3954	[0.3669, 0.4240]	27.64	<0.001
	Condition-mean	0.3954	[0.3656, 0.4252]	27.02	<0.001
CMJA vs. CMJ	Trial-level	0.0592	[0.0499, 0.0685]	12.66	<0.001
	Condition-mean	0.0592	[0.0447, 0.0736]	8.34	<0.001
LCMJ vs. CMJ	Trial-level	−0.0960	[−0.1054, −0.0867]	−20.55	<0.001
	Condition-mean	−0.0960	[−0.1105, −0.0816]	−13.53	<0.001
RFD_250_ (z)	Trial-level	−0.0007	[−0.0321, 0.0308]	−0.04	0.965
	Condition-mean	−0.0007	[−0.0328, 0.0315]	−0.04	0.966
CMJA × RFD_250_	Trial-level	−0.0124	[−0.0220, −0.0028]	−2.58	0.012
	Condition-mean	−0.0124	[−0.0273, 0.0025]	−1.70	0.099
LCMJ × RFD_250_	Trial-level	0.0020	[−0.0076, 0.0116]	0.41	0.682
	Condition-mean	0.0020	[−0.0129, 0.0169]	0.27	0.788

Estimates are expressed on the original jump-height scale in meters, from mixed-effects models with Subject_ID as a random intercept and CMJ as the reference condition. Each model is reported for the trial-level specification (*n* = 102) and the condition-mean specification (*n* = 51). Continuous predictors were standardized as z-scores; their coefficients therefore represent the expected change in jump height associated with a one-standard-deviation increase in the predictor. Degrees of freedom were obtained by Satterthwaite approximation. IMTP: isometric mid-thigh pull; PF: peak force; RFD_250_: rate of force development over the 250 ms epoch; CI: confidence interval.

**Table 5:** Correlations between muscle architecture and IMTP-derived neuromuscular capacity.

Variable	Pearson’s *r* (95% Confidence Limits)	Correlation Description
IMTP RFD_250_		
VL MT	0.57 [0.12, 0.82]	Moderate
VL FL	0.48 [0.00, 0.78]	Moderate
LG PA	−0.05 [−0.52, 0.44]	Negligible
IMTP relative PF		
VL MT	0.20 [−0.31, 0.62]	Weak
VL FL	0.41 [−0.09, 0.74]	Moderate
LG PA	−0.25 [−0.65, 0.26]	Weak

Pearson correlations were calculated from subject-level data (*n* = 17). Confidence limits were calculated using Fisher’s z transformation. The table presents each architecture variable against both IMTP RFD_250_ and relative isometric peak force. The six architecture–RFD_250_ correlations were analyzed as a single pre-specified family with Benjamini–Hochberg false-discovery-rate control; the associations with relative isometric peak force are shown for descriptive comparison. The correlations are therefore interpreted as exploratory. Magnitudes were described as negligible (|*r*| < 0.10), weak (0.10–0.39), moderate (0.40–0.69), strong (0.70–0.89), and very strong (≥0.90). The complete set of six architecture–RFD_250_ correlations, including raw and BH-adjusted *p*-values, is reported in Table S2. VL, vastus lateralis; LG, lateral gastrocnemius; MT, muscle thickness; FL, fascicle length; PA, pennation angle; IMTP, isometric mid-thigh pull; PF, peak force; RFD_250_, rate of force development over the 250 ms epoch.

## Data Availability

Participant-level data are not publicly available because the approved consent did not permit secondary distribution. De-identified data may be considered upon reasonable request and approval from the original ethics committee. The analysis code and a data-free version of the processing workbook may also be provided upon reasonable request, subject to institutional approval.

## Supplementary Materials

The following supporting information can be downloaded at https://www.mdpi.com/article/10.3390/sports14090377/s1: Table S1: Within-condition comparison of the first and second retained trials. Table S2: Correlations between skeletal muscle architecture and isometric RFD_250_ (*n* = 17).
