# Supplementary material for: Arm Swing and External Load Reorganize Force–Time Strategy in Collegiate Basketball Players: Associations with Force Production Capacity
Source: Sports (Basel). 2026 Sep 1;14(9):377. doi: 10.3390/sports14090377 (PMC13611045; doi:10.3390/sports14090377)
Supplement: Supplementary file 1 [file sports-14-00377-s001.zip › sports-4434828-supplementary.pdf]

Supplementary Materials

Arm Swing and External Load Reorganize Force–Time Strategy in Collegiate Basketball Players: Associations with Force Production Capacity

Table S1. Within-condition comparison of the first and second retained trials.

Variable	Condition	Trial 1 (mean ± SD)	Trial 2 (mean ± SD)	Δ (%)	p
Jump height (m)	CMJ	0.40 ± 0.06	0.40 ± 0.06	−0.25	0.712
RSI-mod	CMJ	0.53 ± 0.09	0.52 ± 0.09	−1.46	0.336
Contact time (s)	CMJ	0.755 ± 0.050	0.766 ± 0.070	1.52	0.342
Concentric time (s)	CMJ	0.257 ± 0.022	0.260 ± 0.030	1.19	0.452
Eccentric displacement (cm)	CMJ	39.30 ± 3.63	39.31 ± 5.04	0.02	0.991
Peak force (N)	CMJ	2068 ± 216	2058 ± 221	−0.46	0.550
Mean concentric force (N)	CMJ	1755 ± 177	1748 ± 199	−0.39	0.593
Net impulse (N·s)	CMJ	242.1 ± 30.4	242.1 ± 31.3	−0.01	0.988
Eccentric impulse (N·s)	CMJ	137.7 ± 22.4	134.6 ± 21.1	−2.25	0.049 *
Concentric impulse (N·s)	CMJ	239.7 ± 29.9	239.7 ± 30.8	0.01	0.989
Peak velocity (m·s⁻¹)	CMJ	2.90 ± 0.20	2.90 ± 0.20	0.02	0.957
Take-off velocity (m·s⁻¹)	CMJ	2.78 ± 0.21	2.78 ± 0.21	−0.16	0.717
Vertical stiffness (kN·m⁻¹)	CMJ	52.89 ± 6.11	53.03 ± 7.90	0.27	0.896
Mean concentric power (W)	CMJ	1368 ± 152	1360 ± 196	−0.59	0.679
Peak power (W)	CMJ	4534 ± 606	4540 ± 658	0.13	0.849
Jump rate of force development (N·s⁻¹)	CMJ	7568 ± 2153	7244 ± 2504	−4.28	0.276
Jump time to peak force (s)	CMJ	0.182 ± 0.074	0.204 ± 0.118	12.04	0.342
Jump height (m)	CMJA	0.46 ± 0.07	0.45 ± 0.07	−0.61	0.425
RSI-mod	CMJA	0.52 ± 0.09	0.51 ± 0.09	−2.15	0.043 *
Contact time (s)	CMJA	0.880 ± 0.055	0.895 ± 0.069	1.70	0.057
Concentric time (s)	CMJA	0.291 ± 0.023	0.293 ± 0.026	0.79	0.375
Eccentric displacement (cm)	CMJA	43.61 ± 6.61	44.64 ± 6.15	2.36	0.048 *
Peak force (N)	CMJA	2088 ± 284	2082 ± 293	−0.28	0.770
Mean concentric force (N)	CMJA	1698 ± 210	1692 ± 200	−0.37	0.387
Net impulse (N·s)	CMJA	257.7 ± 33.9	257.6 ± 33.0	−0.05	0.870
Eccentric impulse (N·s)	CMJA	128.3 ± 25.0	131.0 ± 27.6	2.15	0.150
Concentric impulse (N·s)	CMJA	254.9 ± 33.3	254.9 ± 32.8	−0.02	0.940
Peak velocity (m·s⁻¹)	CMJA	3.10 ± 0.23	3.10 ± 0.23	−0.08	0.809
Take-off velocity (m·s⁻¹)	CMJA	2.98 ± 0.23	2.97 ± 0.24	−0.32	0.415
Vertical stiffness (kN·m⁻¹)	CMJA	48.80 ± 9.62	47.29 ± 8.49	−3.10	0.138
Mean concentric power (W)	CMJA	1385 ± 226	1373 ± 222	−0.84	0.359
Peak power (W)	CMJA	5287 ± 772	5222 ± 788	−1.23	0.058
Jump rate of force development (N·s⁻¹)	CMJA	3623 ± 1940	3861 ± 2724	6.57	0.261
Jump time to peak force (s)	CMJA	0.397 ± 0.099	0.393 ± 0.110	−0.96	0.592
Jump height (m)	LCMJ	0.30 ± 0.04	0.30 ± 0.04	−1.68	0.046 *
RSI-mod	LCMJ	0.37 ± 0.06	0.36 ± 0.06	−1.76	0.310
Contact time (s)	LCMJ	0.828 ± 0.048	0.830 ± 0.054	0.28	0.812
Concentric time (s)	LCMJ	0.301 ± 0.022	0.300 ± 0.026	−0.22	0.885
Eccentric displacement (cm)	LCMJ	39.81 ± 3.57	40.08 ± 4.07	0.67	0.696
Peak force (N)	LCMJ	2129 ± 242	2110 ± 219	−0.89	0.301
Mean concentric force (N)	LCMJ	1845 ± 197	1843 ± 200	−0.13	0.865
Net impulse (N·s)	LCMJ	259.5 ± 31.2	258.0 ± 31.3	−0.58	0.116
Eccentric impulse (N·s)	LCMJ	156.7 ± 23.9	158.4 ± 27.1	1.06	0.417
Concentric impulse (N·s)	LCMJ	256.0 ± 30.8	254.1 ± 30.6	−0.73	0.066
Peak velocity (m·s⁻¹)	LCMJ	2.57 ± 0.15	2.55 ± 0.16	−0.71	0.059
Take-off velocity (m·s⁻¹)	LCMJ	2.43 ± 0.17	2.41 ± 0.18	−0.84	0.040 *
Vertical stiffness (kN·m⁻¹)	LCMJ	53.79 ± 6.85	53.05 ± 6.77	−1.37	0.465
Mean concentric power (W)	LCMJ	1115 ± 173	1104 ± 179	−1.02	0.593
Peak power (W)	LCMJ	4392 ± 622	4362 ± 612	−0.69	0.313
Jump rate of force development (N·s⁻¹)	LCMJ	5673 ± 1570	5404 ± 1468	−4.75	0.135
Jump time to peak force (s)	LCMJ	0.220 ± 0.084	0.227 ± 0.084	3.24	0.044 *
RFD (0–250 ms) (N·s⁻¹)	IMTP	6616 ± 1770	6627 ± 1884	0.16	0.938
Isometric peak force (N)	IMTP	3199 ± 615	3169 ± 652	−0.93	0.280

Within each condition, the first and second retained trials were compared using paired-samples t tests (n = 17). Δ (%) = (Trial 2 − Trial 1) / Trial 1 × 100. Jump height was calculated from take-off velocity. Six of the 53 comparisons reached statistical significance, all with small changes (|Δ| ≤ 3.3%) and inconsistent in direction. CMJ, countermovement jump; CMJA, countermovement jump with arm swing; LCMJ, loaded countermovement jump; IMTP, isometric mid-thigh pull; RSI-mod, reactive strength index-modified; RFD, rate of force development; SD, standard deviation. * $p < 0.05$.

Table S2. Correlations between skeletal muscle architecture and isometric RFD_{250} (n = 17).

Muscle site	Variable	r [95% CI]	Raw p	BH-adjusted p
Vastus lateralis	Muscle thickness	0.570 [0.123, 0.825]	0.017	0.101
	Fascicle length	0.478 [−0.004, 0.779]	0.052	0.157
	Pennation angle	0.156 [−0.351, 0.592]	0.550	0.995
Lateral gastrocnemius	Muscle thickness	−0.023 [−0.498, 0.463]	0.931	0.995
	Pennation angle	−0.049 [−0.518, 0.442]	0.851	0.995
	Fascicle length	0.002 [−0.479, 0.482]	0.995	0.995

BH, Benjamini–Hochberg. All six correlations of the pre-specified family are reported. Fascicle length was derived from muscle thickness and pennation angle, and is therefore mathematically dependent on them.
